# Impact of Vaping Regimens on Electronic Cigarette Efficiency

**DOI:** 10.3390/ijerph16234753

**Published:** 2019-11-27

**Authors:** Sébastien Soulet, Marie Duquesne, Jean Toutain, Charly Pairaud, Maud Mercury

**Affiliations:** 1 Ingesciences, 2 Chemin des Arestrieux, 33610 Cestas, France; 2 Bordeaux University, CNRS, I2M Bordeaux, Esplanade des Arts et Métiers, F-33405 Talence CEDEX, France; charly.pairaud@ingesciences.fr (C.P.); maud.mercury@ingesciences.fr (M.M.); 3 Bordeaux INP, CNRS, I2M Bordeaux, ENSCBP, 16 Avenue Pey Berland, 33607 Pessac CEDEX, France; marie.duquesne@enscbp.fr (M.D.); jean.toutain@enscbp.fr (J.T.)

**Keywords:** inhalation properties, vaping regimen, electronic cigarette efficiency, mass of vaporized e-liquid, puff duration, puff volume

## Abstract

Most recent studies on electronic cigarettes (e-cigs) have been carried out using vaping regimens consistent with mouth-to-lung inhalation (MTL) and not with direct-to-lung (DTL) inhalation. This paper aimed to characterizing the influence of inhalation properties (puff duration, puff volume, airflow rate) on the mass of vaporized e-liquid (MVE). Because the literature on DTL is non-existent, an intense vaping regimen consistent with DTL inhalation (i.e., puff volume = 500 mL) was defined. The use of a low or standard (ISO/DIS 20768) regimen and the proposed intense vaping regimen were first compared using the Cubis 1 Ω atomizer on a large power range, and then by using two atomizers below 1 Ω and two others above 1 Ω on their respective power ranges. An analysis of the e-cig efficiency on the e-liquid vaporization was proposed and calculated for each MVE. The intense vaping regimen allowed a broader power range in optimal heating conditions. MVE linearly increased with the supplied power, up to over-heating conditions at higher powers. Moreover, the e-cigs’ efficiencies were higher when low-resistance atomizers were tested at high powers. All these results highlighted that the generated vapor might be better evacuated when an intense vaping regimen is used, and illustrate the obvious need to define a suitable standardized vaping regimen consistent with DTL inhalation.

## 1. Introduction

Since electronic cigarettes (e-cigs) first appeared on the market, their designs have evolved and e-cigs no longer look like classical cigarettes (cig-like products). They are mainly composed of a battery, a resistive wire, a fibrous media (wick), a multi-component liquid mixture (e-liquid), and a tank for e-liquid. The wire transforms electrical energy into thermal energy as soon as the battery is activated and heats the surrounded e-liquid. E-cigs have holes for air entrance during inhalation. The air passing through these holes is then mixed with the vapor generated when the e-liquid is vaporized. E-cigs are commonly divided into three groups [1]: first-, second-, and third-generation devices, depending on their design. In 2015, in Europe, first- and second-generation devices were used, respectively, by 16% and 35% of e-cig users, while third-generation e-cigs were used by 62% of consumers (some consumers used e-cigs of both the second and third generations) [2]. Third-generation e-cigs are opened systems with large holes (with a wide range of diameters that are fixed or adjustable) for air entrance; hence, they generate a low resistance during inhalation. This low resistance leads to a lower inhalation effort. This wide range of designs leads to a higher range of resistances during inhalation from one device to another. Until now, there has been no publication dealing with measurement of the resistance induced by e-cigs during inhalation. Anecdotally, users feel that inhaling through an e-cig requires less effort than through a classical cigarette. Consequently, users will have inhalation profiles (or puff behavior, puff profile, puff topography) closer to breathing. This resistance diversity in e-cig allows a wide type of inhalation profiles. Two main behaviors are commonly defined: mouth-to-lung (MTL) and direct-to-lung (DTL) inhalation. In MTL inhalation, the user keeps the vapor in his mouth, stops vaping, and inhales air in order to dilute and move the vapor to his lungs. In DTL inhalation, the user inhales as he breathes. There is no inhalation stop and the vapor goes directly into the lungs. Physically, such differences are directly linked to the puff duration and inhaled volume (and consequently to the airflow rate). An initial estimation of the puff volume considered the MTL puff volume to be closer to the mouth volume (55 mL) [3] and the DTL puff volume to be closer to the volume commonly inhaled by humans. Medically, this volume is called the human tidal volume (500 mL) [4].

Most existing studies about puff behavior have been carried out with cig-like or first-generation [5,6,7,8,9,10] and second-generation [11,12] e-cigs, giving puff volumes between approximately 50 mL and 100 mL (corresponding to an MTL inhalation profile) and puff durations between 2 s and 4 s. In other words, the available studies reporting on puff behavior represent only approximately half of e-cig users and types of e-cigs. Current e-cig research is mostly focused on the qualification and quantification of emissions (chemical analysis [13,14,15,16,17,18,19], particle size [20,21,22,23,24], etc.). Emissions are generated using a vaping machine configured with a protocol composed of a puff duration, a puff volume, an airflow rate, and a puff frequency. This protocol is commonly referred to as a “vaping regimen” or “puffing regimen”. In a recent publication [25], the authors reviewed different vaping regimens. Over the 20 papers available in the literature, almost as many vaping regimens have been used. Such diversity does not allow for comparison of results. 

Since 2015, national standards have been published [26,27,28,29] aiming to standardize e-cigs, e-liquid manufacturing, and analyses of the generated emissions. In 2018, an international standard ISO/DIS 20768 [30] was published dealing with vaping machines and a standard vaping regimen. Based on CORESTA (Cooperation Centre for Scientific Research Relative to Tobacco) recommended method 81 [31], this standard defined a standard puff duration of 3 s, a puff volume of 55 mL, and a puff period of 30 s. This puff volume is characteristic of smoking [32], and an MTL inhalation profile was applied to an intense smoking regimen [33,34]. However, it is not representative of DTL inhalations. Due to technical limitations of the smoking machine [35], it was not upgraded to be more consistent. This standard vaping regimen is referred as the “low vaping regimen” in the rest of this paper.

A few publications have tried to characterize the influence of vaping regimen on the emissions of first- [22,36], second- [37], and third-generation e-cigs [25,38]. For this last group of e-cigs, a longer puff duration and a longer puff volume increases the mass of vaporized e-liquid (MVE). Generally, production of aldehydes such as formaldehyde, acetaldehyde, and acrolein also increases when emissions are generated using higher inhalation conditions. Regarding this observation, the results presented when a low vaping regimen is used to characterize a third-generation atomizer [39,40,41,42] would probably be significantly different with the use of an intense vaping regimen. 

This paper aimed to characterize the influence of inhalation characteristics on MVE. A single e-liquid, referred as the reference e-liquid, was vaporized in all the presented results. The first series of experiments consisted of varying the puff duration with either a constant airflow or with a constant puff volume. Puff durations from 1 s to 10 s were tested with a constant airflow of 18.3 mL·s^−1^ (corresponding to a constant puff volume of 55 mL). Puff volume influence was then investigated, keeping the puff duration constant. To do so, a Cubis atomizer (Joyetech, Shenzhen, China) with its 1 Ω coil (rolled wire) was used as a reference atomizer at a fixed power. Puff volume was tested from 25 mL to 500 mL. 

**Figure 1:**
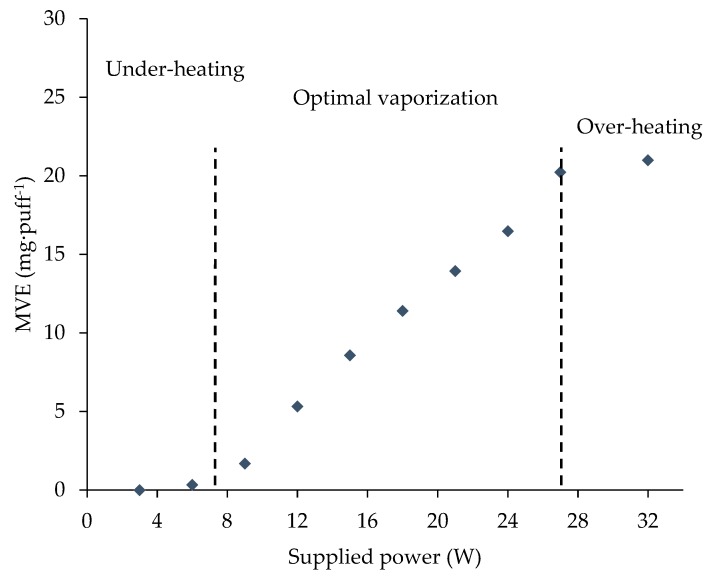
Description of the three heating regimens (supplied power versus mass of vaporized e-liquid (MVE))—example for the Cubis 1 Ω atomizer.

Secondly, low and intense vaping regimens were compared. They differed in puff volumes, which were set at, respectively, 55 mL and 500 mL, corresponding to the mouth volume (standardized puff volume) and tidal volume. In a recent publication, we illustrated three heating regimens (Figure 1): an under-heating regimen at low power with no generated vapor, an optimal regimen characterized by a MVE proportional to the supplied power, and an over-heating regimen [43]. The same experiment was carried out with an intense vaping regimen (3 s of puff duration, 500 mL of puff volume, 166.7 mL·s^−1^ of puff airflow) in order to observe its influence on these three heating regimens. The Cubis 1 Ω atomizer was thus tested through a large power range up to the over-heating regimen.

Finally, the intense vaping regimen was used with four other atomizers: two with low electric resistances (0.5 Ω) and two with high electric resistances (1.5 Ω and 1.8 Ω). These atomizers were tested in the power range specified by their manufacturers. The presented experiments aimed to compare their MVEs, previously obtained using a low vaping regimen (3 s of puff duration, 55 mL of puff volume, 18.3 mL·s^−1^ of puff airflow) [43], with those recorded under the intense vaping regimen (3 s of puff duration, 500 mL of puff volume, 166.7 mL·s^−1^ of puff airflow). An analysis of e-cig efficiency was proposed based on the MVE measurement. The impact of the vaping regimen on the five atomizers is discussed below.

## 2. Materials and Methods

### 2.1. Reference E-Liquid

Commercial e-liquids mainly contain a basis of propylene glycol (PG) and glycerol (VG). Water (H_2_O) and ethanol (EtOH) may also be added in lower proportions. Nicotine and flavors can then also be added in lower concentrations. In Soulet et al. (2018), a study of the influence of power on MVE for several atomizers was performed using a low vaping regimen [43]. Here, we aimed to study the influence of the vaping regimen on MVE. Hence, the same reference e-liquid was used to allow comparison with the previously published results. The liquid used was composed of the pure molecules listed in Table 1.

The volume, mass, molar compositions, and main key properties of these molecules are listed in Table 2. 

### 2.2. Vaping Machine and Vaping Regimens 

The tests were performed using a U-SAV (Universal System for Analysis of Vaping) vaping machine [44]. The protocol was based on the AFNOR (French Association for Standardization) protocol [28]. The repeatability of MVE over series was previously illustrated in Soulet et al. (2018) [43]; therefore, the protocol was shorter and experiments were composed of only two series of 20 puffs per series. Series were separated by a 5 minute inter-series break. Atomizer inclination was set at 45° with respect to the vertical position during the vaporization process, and moved back to 0° over 10 s in the inter-series break, as defined in the AFNOR standard [28].

In the ISO (International Organization for Standardization) 20768 standard [30], the vaping regimen is defined by a puff duration of 3 s, a puff volume of 55 mL, a rectangular shape puff profile with a constant airflow rate of 18.3 mL·s^−1^, and a puff period of 30 s. This regimen is representative of a low vaping regimen. The obtained MVEs were compared to those obtained using an intense vaping regimen. The latter was characterized by a puff volume of 500 mL with a puff duration of 3 s and a rectangular shape puff profile, leading to a constant airflow rate of 166.7 mL·s^−1^.

### 2.3. Atomizers

Five commercial atomizers were tested: Cubis 1 Ω (Joyetech, Shenzhen, China), Mini-C 0.5 Ω (Kangertech, Shenzhen, China), I-Sub 0.5 Ω (Innokin, Shenzhen, China), Nautilus 1.8 Ω (Aspire, Shenzhen, China), and GS Air 1.5 Ω (Eleaf, Shenzhen, China). Each commercial atomizer was tested in three replicates in order to measure the variability over the devices. The main information regarding the atomizers is provided in Table 3.

Due to the short recommended range of the Nauti 1.8 Ω coil, it was extended from 3.3 V to 6 V, which is the recommended range of the Nauti 1.6 Ω coil, leading to a power range of 6.05 W to 20 W. In a recent publication [45], the vaporization of pure PG, VG, and EtOH was studied using the Cubis 1 Ω atomizer. It was shown that the supplied power of 15 W was consistent with the optimal heating regimen observed for these three pure components. This supplied power was here considered as the reference power for this atomizer. The repeatability of the Cubis 1 Ω atomizer was shown in Soulet et al. (2018) [43].

### 2.4. Experiments

Atomizers were filled with the reference e-liquid (Table 2) up to the maximum line specified by the manufacturer. The influence of inhalation properties was characterized using the MVE by weighting the tested atomizer before and after each experiment, and then the global MVE was determined by computing the difference between the two masses. This value was finally divided by the number of puffs during the experiment. The Mettler AT261 DeltaRange laboratory scale allowed the measurement of masses ranging from 1 mg to 205 g with a precision of 0.1 mg. 

First, the influences of puff duration and puff volume on the MVE were characterized using the Cub1 atomizer at a fixed power of 15 W. Puff duration was investigated in two ways from 1 s to 10 s with a step of 1 s. The puff duration was first tested with a fixed puff volume of 55 mL, and then with a fixed airflow rate of 18.3 mL·s^−1^. Subsequently, puff volume was tested from 0 mL to 500 mL with a step of 25 mL and a puff duration fixed at 3 s. 

Secondly, we aimed to characterize the heating regimens (Figure 1) when the intense vaping regimen was applied using the Cub1 atomizer. Therefore, the supplied power was increased until over-heating was reached. A large power range was tested. The results were compared to the results of the experiments carried out with the low vaping regimen [43]. 

Finally, focusing only on the optimal heating regimen, the influence of the intense vaping regimen was tested on the other atomizers. For each atomizer, five supplied powers were selected and applied. They were equally distributed in their respective power ranges specified by the manufacturer. The MVEs obtained were compared to those measured using a low vaping regimen in Soulet et al. (2018) [43].

## 3. Results

### 3.1. Influence of Inhalation Properties Using Cub1 Reference Atomizer at a Fixed Power of 15 W

#### 3.1.1. Influence of Puff Duration at a Fixed Puff Volume of 55 mL and at a Fixed Air-Flow Rate of 18.3 mL/s

**Figure 2:**
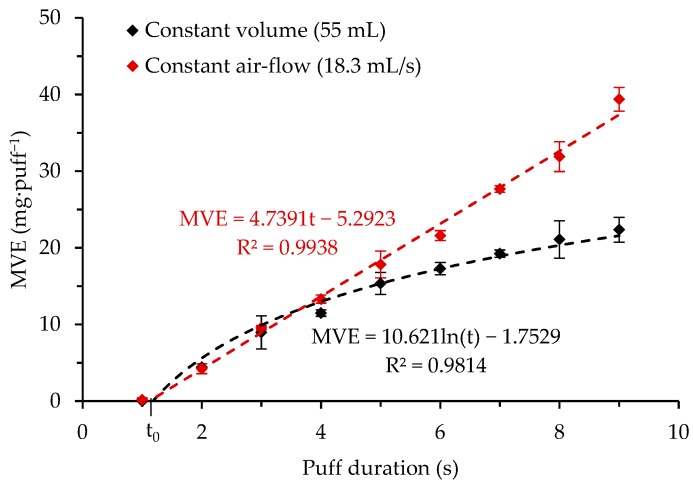
MVE—influence of puff duration while keeping puff volume constant (dark dots) and airflow rate constant (red dots) for the Cub1 reference atomizer at 15 W.

Figure 2 illustrates the influence of the puff duration on the MVE. Fixing a puff volume and increasing the puff duration automatically led to an airflow rate reduction. The puff duration influence was first characterized with a constant airflow rate of 18.3 mL·s^−1^, and then with a constant puff volume of 55 mL. In the first case, the measured MVE followed a linear trend with a determination coefficient of 0.9938. This linear trend revealed that the MVE remained constant second by second, even with long puff duration, while the airflow was constant. When a fixed volume was maintained, MVEs were also increased with increasing puff durations. In contrast to the constant airflow rate, this trend had a logarithmic profile. Comparing the two experiments, at short puff duration (<3 s), a difference between the two conditions was not evident. Above a 3 s puff duration, the difference became significant and continued to grow as the puff duration increased. 

Furthermore, by extending the linear trends to the abscissa axis, a characteristic time (noted t_0_ in Figure 1) could be observed, revealing that a time lapse is needed for e-liquid vaporization (i.e., MVE > 0 mg·puff^−1^). At 15 W, t_0_ was 1.12 s for the red line and 1.18 s for the black one.

#### 3.1.2. Influence of Puff Volume at a Fixed Puff Duration of 3 s

**Figure 3:**
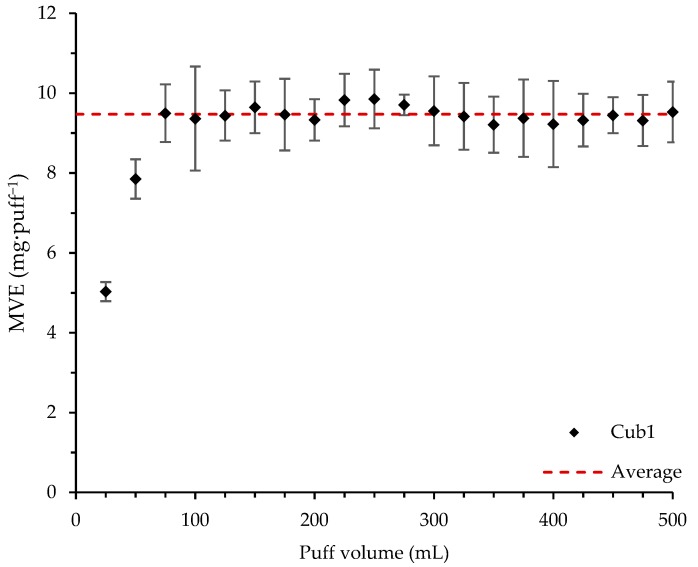
MVE—puff volume influence with constant puff duration constant (3 s) using the Cub1 reference atomizer with a 15 W supplied power.

Figure 3 reports the puff volume’s influence on MVE using the Cub1 reference atomizer. MVE had a non-linear trend. For puff volumes ranging from 0 mL to 50 mL, MVE increased with puff volume, after which MVE stayed constant at around 9.47 mg·puff^−1^ ± 0.19 mg·puff^−1^. As observed in the previous part, at low puff volumes, the airflow rate was also reduced. These results suggest that there is an adequate puff volume (i.e., airflow rate) that must be applied to evacuate all the generated vapor. Here, this volume as close to 75 mL.

### 3.2. Comparison of Results Obtained Using Low and Intense Vaping Regimens with Cub1 Reference Atomizer

**Figure 4:**
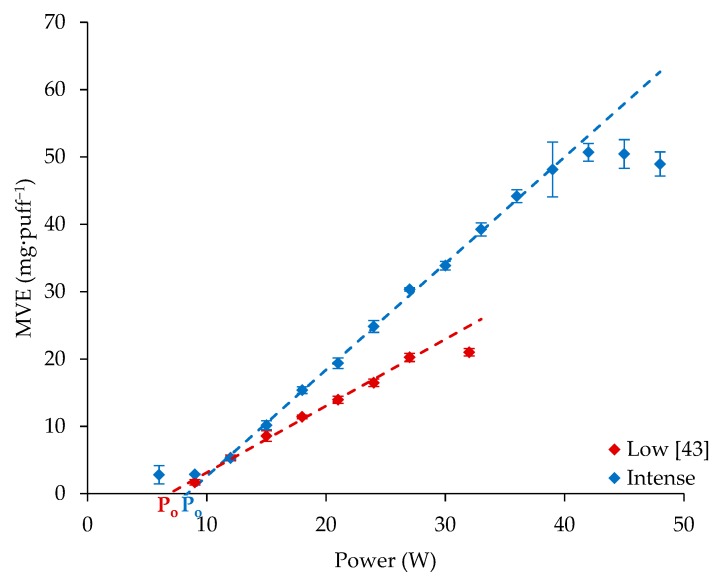
MVE—comparison between the low and intense vaping regimens’ MVE for the Cub1 reference atomizer (P_0_, minimal power).

Figure 4 illustrates the influence of the supplied power on MVE using the low and intense vaping regimens. MVE measured when the intense profile (blue dots) was applied was then compared with the previously published results obtained when a low regimen was used [43] (red dots). When the intense regimen was applied, an optimal regimen (linear trends with a determination coefficients upper than 0.99, Table 4) was observed from 9 W to 39 W. Comparatively higher puff volumes allowed higher MVE and wider optimal regimen ranges. Here, the slope was increased by 59% between the intense and standard regimens. After 39 W, an over-heating regimen was also observed for the intense regimen—this occurred 12 W above that found for the low regimen (27 W).

Furthermore, from the linear trend of the optimal regimen, a characteristic power could be extracted (noted P_0_ in Figure 3 and Table 4 and Table 5), representing the minimal power that has to be supplied to activate the transition from liquid to vapor. This could also be calculated by dividing the intercept point coefficient (b) and the slope coefficient (a). This minimal power increased with the use of an intense regimen (i.e., a higher puff volume), suggesting that the coil temperature’s rising slowed when high puff volumes were used. 

### 3.3. Comparison of the Results Obtained Applying the Low and Intense Vaping Regimens Using Different Atomizers

**Figure 5:**
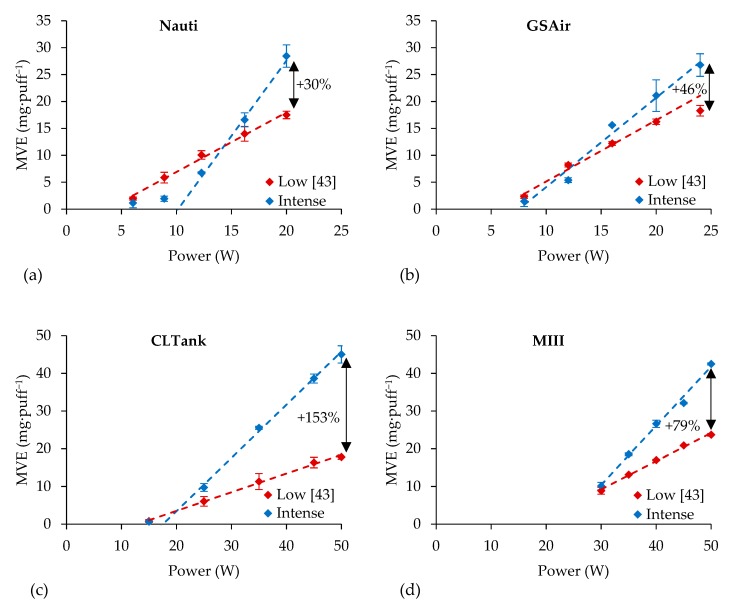
MVE—comparison between the low and intense regimens on the (**a**) Nauti, (**b**) GS, (**c**) CLTank, and (**d**) MIII atomizers over their respective power ranges.

The MVEs of the other four atomizers tested in their recommended power ranges (determined by the manufacturers) using the intense vaping regimen are illustrated in Figure 5. The measured MVEs were compared to the ones obtained using a low vaping regimen in Soulet et al. (2018) [43].

At low supplied powers, the Nauti, GS, and CLTank, respectively, had 3, 2, and 1 measurements lower when using the intense regimen than with the low vaping regimen. All MVEs were higher. The MVEs of MIII were strictly superior to those obtained using the low vaping regimens. At their highest tested powers, the MVEs were, respectively, 46%, 63%, 79%, and 153% higher for the GS, Nauti, MIII, and CLTank atomizers with the intense vaping regimen than with the low one. 

With regard to the supplied power, each atomizer had rather linear MVEs (omitting the few MVEs that were out of the optimal heating regimen). The linear trend slopes, intercept points and determination coefficients were extrapolated and reported in Table 5. The ones obtained using a low vaping regimen [43] were also added in Table 5. The determination coefficients were higher than 0.99 (except the Nauti, which did not have enough MVE above zero), revealing that the optimal heating regimens were maintained despite the use of the intense vaping regimen. As for the Cub1, the initial powers that had to be supplied to see an MVE higher than 0 mg·puff^−1^ (observable with the linear trends) were increased with the use of the intense vaping regimen (i.e., with the puff volume increasing). This was observable at low supplied powers for the Nauti and CLTank atomizers with the MVE out of the optimal heating regimen.

### 3.4. Impact of Inhalation Vaping Regimen on E-Cig Efficiency

In the drying process, the energy performance of a dryer could be evaluated using the technical specification of energy efficiency [46]. Energy efficiency expresses the ratio between the energy used for moisture evaporation and the supplied energy. By extrapolation, for e-cig applications, the e-cig efficiency (η) could be determined as the ratio between the energy used to heat and vaporize the measured MVE and the energy supplied by the battery, following Equation (1).

(1) $$\eta =\frac{\mathrm{MVE}\cdot \left(C_{p}\cdot \Delta T_{s}+H^{v}\right)}{P\cdot \Delta t}.$$

where H^v^ (J·g^−1^) and C_p_ (J·K^−1^·g^−1^) are the enthalpy of vaporization and heat capacity of the e-liquid, respectively; ∆T_s_ (K) is the difference between the specific and ambient temperatures; P (W) is the supplied power; and ∆t (s) is the puff duration. The closer this ratio is to 100%, the less energy is lost and the more supplied energy is used to heat and vaporize the e-liquid. Considering that the liquid and vapor compositions are very close [47], the specific temperature corresponds to the dew point temperature (T_r_) of the e-liquid. Its determination was made using the non-random two-liquids (NRTL) semi-predictive model [48] for vapor–liquid equilibrium determination. T_r_ was found to be equal to 533.8 K. Here, the puff duration was 3 s. An estimation of the mass heat capacity (C_p_) and enthalpy of vaporization (H_v_) for the tested e-liquid (composed of n pure components) was calculated using Equations (2) and (3).

(2) $$H_{v}=\frac{\sum_{i}^{n}x_{i}h_{i}^{v}}{\sum_{i}^{n}x_{i}M_{i}}$$

(3) $$C_{p}=\frac{\sum_{i}^{n}x_{i}c_{p}{}_{i}}{\sum_{i}^{n}x_{i}M_{i}}$$

where h^v^_i_ (J·mol^−1^), c_pi_ (J·K^−1^·mol^−1^), M_i_ (g·mol^−1^), and x_i_ represent, respectively, the heat capacity, the enthalpy of vaporization, the molar mass, and the mole percent of component i. The reference e-liquid used has an estimated heat capacity of 2.42 J·K^−1^·g^−1^ and an enthalpy of vaporization of 934 J·g^−1^, using Equations (2) and (3). The efficiencies of the five atomizers using the low and intense vaping regimens were calculated and are reported in Table 6. (A dash means that the MVE was out of the optimal heating regimen and was not considered.)

For each atomizer, its efficiency of e-liquid vaporization increased with the supplied power. For the Cub1, MIII, and CLTank atomizers, the use of an intense regimen significantly increased their efficiencies compared to the use of a low vaping regimen. This observation was also verified for Nauti and GSAir atomizers when high powers were supplied.

## 4. Discussion

The results illustrated and highlighted these points: 

At 15 W on the Cub1 atomizer, maintaining a constant air flow, MVE linearly increased with the puff duration, whereas maintaining a constant puff volume led to a MVE that followed a logarithmic growth pattern. A latency time to see MVE rising above 0 mg·puff^−1^ was identified.

A rectangular shape puff profile by definition leads to a constant airflow. Therefore, the linear increase in MVE implied a rectangular shape puff profile in the standard protocol. Considering the aerosol as having the same composition as the e-liquid, this suggests that the same quantity of each component is vaporized in the given time. The Tobacco Product Directive [49] requires a constant nicotine delivery. Although the official definition is under discussion, a constant nicotine delivery can be defined as the repeatability of an atomizer delivering the same quantity of nicotine in a given time. Following the previous observations, this requirement would be automatically checked until an atomizer was used in optimal regimen conditions. 

At 15 W on the Cub1 atomizer, keeping the puff duration constant, MVE appeared to have an average value of 9.43 mg·puff^−1^ ± 0.26 mg·puff^−1^. Below a volume limit of 75 mL for the Cub1 atomizer, MVEs were lower than this average value. 

At low airflow rates, the generated vapor was not sufficiently evacuated, leading to under-estimation of MVE. This could be problematic when atomizers are developed and tested in order to characterize their efficiency and/or the quantity of nicotine vaporized per puff. Under this condition, a part of the vapor will remain in suspension or condensate on the wall part of the atomizer. This condensed vapor will form droplets that will return to the coil and will be finally vaporized again. In the end, a mass of e-liquid will be decomposed, as vaporization will occur many times. For the part in suspension, the vapor is sustained at the e-liquid dew point temperature until the coil heats up. Thus, PG and VG decompositions [50,51] will be maintained. Therefore, some byproducts will be generated if an unsuitable protocol regarding the tested atomizer is applied.

The low and intense vaping regimens gave different results. For the Cub1 atomizer, significant observations were made over the three heating regimens. During the under-heating regimen, an increase in the minimal power required to see vapor generation was observed. During the optimal regimen, MVE was significantly increased compared to the low vaping regimen. Furthermore, the power range characterizing the optimal regimen was considerably larger, and the over-heating regimen occurred at higher supplied powers. The four other atomizers tested also presented an increase in the minimal power that had to be supplied to see generated vapor. For the Nauti, GSAir, and CLTank atomizers, this led to an observable latency time before the beginning of the optimal regimen, resulting in a lower MVE with an intense vaping regimen than with a low vaping regimen at low supplied powers. At high supplied power, MVE was significantly superior using an intense vaping regimen compared to a low one. This was even more noticeable when low-resistance atomizers were used. 

The minimal power (needed to activate the vaporization) showed a minimal required energy to heat the system from ambient temperature to boiling. This energy is linked to the thermal inertia of the coil and explains why the tested atomizers have different minimal powers. The use of an intense vaping regimen resulted in a higher flowrate of the air inside the atomizer, leading to higher heat exchanges by forced convection from the coil to the air. These heat exchanges dampened the temperature rise of the coil. Therefore, reaching the boiling temperature of the liquid will require more energy in an intense vaping regimen than in a low one. This was observed for the Nauti, GSAir, and CLTank atomizers at low supplied powers. MVE and the resulting efficiencies were lower in the intense vaping regimen than in the low one. This was due to the supplied energy not being sufficient to overcome the influence of the air. 

However, the calculated efficiencies were higher when high powers were coupled with intense vaping regimens. This was even more noticeable when high-resistances atomizers were used, with efficiencies over 50% reached. This suggests that the intense vaping regimen is more appropriate for these atomizers, and could even be increased for sub-ohm atomizers (atomizers with a resistance lower than 0.5 Ω). DTL inhalation could also be defined using a puff volume considerably higher than the tidal volume: the inspiratory capacity volume (IC, corresponds to the amount of air inhaled during a forced breath) of 3600 mL [52]. This extrema vaping regimen could be more appropriate for users who make a forced inhalation, e.g., people doing cloud chasing. CLTank and MIII atomizers would have even higher MVEs, leading to efficiencies higher than 50%, using this extreme vaping regimen. 

Generally, when a low regimen is applied in a third-generation atomizer, emissions are not correctly evacuated. All these observations illustrate the obvious need to define at least a suitable standardized vaping regimen consistent with DTL inhalation, and a second one consistent with extreme DTL inhalation. The proposed protocol has the same puff duration as the standard one, but the puff volume is now proposed to be equal to a physical value: the tidal volume (500 mL). 

## 5. Conclusions

This paper focused on the influence of inhalation properties on MVE. A reference liquid (0.2% of Nico, 10% of EtOH, 44.8% of PG, and 45% of VG by volume), was used. A Cub1 atomizer was first used as a reference, and then four others were tested (each from a different manufacturer). Two atomizers had electric resistances higher than 1 Ω and two below. First, puff volume and puff duration influences were characterized. At a fixed airflow rate, puff duration linearly (R² = 0.9938) increased MVE, whereas fixing a puff volume (R² = 0.9814) increased MVE logarithmically. At a fixed puff duration and low puff volume, MVE increased until puff volume reaches a limit at which MVE stayed constant (9.47 mg·puff^−1^). Finally, an intense vaping regimen was applied. Important differences were observed between the MVE measured in these conditions and using a low vaping regimen. Using the Cub1 atomizer with an intense vaping regimen allowed a larger range of optimal heating regimen (9 W–39 W versus 10 W–27 W) where the MVE linearly increased with the supplied power. The steepness of the slope was also increased (1.58 mg·puff^−1^ versus 0.99 mg·puff^−1^). This led to an over-heating regimen appearing at a higher power and MVE. A definition of e-cig efficiency was proposed, considering that to be efficient, an e-cig should use all the supplied energy to bring the liquid from ambient conditions to its vaporization. The higher the efficiency value, the less energy will be lost in the atomizer. The calculated efficiencies were significantly higher when the atomizers were tested using the intense vaping regimen at high powers. 

With the use of a low and an intense vaping regimen, we illustrated the different results that could be obtained between MTL and DTL inhalations. We recommend not using the available standardized vaping regimen for low-resistance atomizers (especially at high powers) due to the risk of publishing results inconsistent with real use. Furthermore, there is an urgent need to define, at minimum, a suitable standardized vaping regimen consistent with DTL inhalation. 

With the last series of experiments, combined with the previously presented results [43,45], we more precisely characterized the influence of the main physical parameters involved on the Cub1 atomizer. For some parameters, additional tests were carried out on the other atomizers. Future ΖIn view of the inexistent literature on DTL, puff volume, puff duration, and airflow rates of vapers inhaling the vapor directly into their lungs must be measured.

- The choice to use an intense instead of a standardized vaping regimen must be linked to a criterion of application. This criterion would be a technical characteristic of the e-cig and would allow a group of products to be defined. The inhalation resistance generated by the atomizers would be an interesting property that could lead to more or less inhalation effort by the users. Future experiments will be carried out on the measurement of atomizers’ resistance during inhalation.
- E-cig efficiencies were found to be lower than 100%, revealing energy lost in the tested atomizers. An identification and quantification of where energy is lost in an e-cig must be performed, and would allow a better understanding of the heat transfers occurring in an e-cig.

## Figures and Tables

**Table 1:** List of the studied pure liquids and general information.

Pure Liquids	Acronym	CAS Number	Formula	Provider	Purity (%)
Nicotine	Nico	54-11-5	C_10_H_14_N_2_	ALCHEM	≥99.2%
Ethanol	EtOH	64-17-5	C_2_H_6_O	GROSSERON	96%
Propylene glycol	PG	57-55-6	C_3_H_8_O_2_	BRENNTAG	≥99.8%
Glycerol	VG	56-81-5	C_3_H_8_O_3_	AMI CHIMIE	99.5%

**Table 2:** Composition and properties of the studied liquid at ambient temperature (Design Institute for Physical Properties (DIPPR) database: https://www.aiche.org/dippr).

Quaternary Mixtures	Volume Percent (%)	Density (g·cm^−3^)	Mass Percent (%)	Molar Mass (g·mol^−1^)	Mole Percent (%)	Molar Heat Capacity (J·mol^−1^·K^−1^)	Molar Enthalpy of Vaporization (kJ·mol^−1^)
Nico	0.20	1.01	0.18	162.24	0.09	-	56.60
EtOH	10.00	0.79	7.09	46.07	12.24	110.46	42.85
PG	44.80	1.04	41.83	76.10	43.71	188.59	66.98
VG	45.00	1.26	50.90	92.09	43.96	219.39	90.21

A dash means that the value is not available.

**Table 3:** Manufacturers’ general information about the studied atomizers.

Manufacturer	Reference	Resistance	Metal	Wick	Notation	Min	Max
Joyetech	Cubis	1 Ω	SS316L	Organic cotton	Cub1	10 W	25 W
Kangertech	CL Tank	0.5 Ω	SS316L	Organic cotton	CLTank	15 W	60 W
Eleaf	Melo III	0.5 Ω	Kanthal	Organic cotton	MIII	30 W	100 W
Aspire	Nautilus	1.8 Ω	Kanthal	Cotton	Nauti	4.2 V (10 W)	5 V (14 W)
Eleaf	GS Air	1.5 Ω	Kanthal	Organic cotton	GS	8 W	20 W

**Table 4:** MVE—values of the coefficients a and b and their standard deviations (Δa) and (Δb) in the equation MVE = aP + b using the Cub1 atomizer and the low and intense vaping regimens (R^2^ = determination coefficient).

Vaping Regimen	a (mg·W^−1^·puff^−1^)	Δa (mg·W^−1^·puff^−1^)	b (mg·puff^−1^)	Δb (mg·puff^−1^)	*R* ^2^	P_0_ (W)
Low [43]	0.99	0.03	−6.76	0.52	0.9962	6.81
Intense	1.58	0.03	−13.20	0.91	0.9965	8.35

**Table 5:** MVE—values of the coefficients a and b and their standard deviations (Δa) and (Δb) in the equation MVE = aP + b using the other atomizers and the low and intense vaping regimens (R^2^ = determination coefficient).

Vaping Regimen	Device Acronyms	a (mg·W^−1^·puff^−1^)	Δa (mg·W^−1^·puff^−1^)	b (mg·puff^−1^)	Δb (mg·puff^−1^)	*R* ^2^	P_0_ (W)
Low [43]	Nauti	1.11	0.05	−4.16	0.71	0.9934	3.75
	GS	1.14	0.08	−6.27	1.16	0.9906	5.50
	CLTank	0.52	0.00	−6.94	0.12	0.9999	13.35
	MIII	0.75	0.03	−13.14	1.21	0.9913	17.52
Intense	Nauti	2.80	0.18	−28.40	2.92	0.9960	10.14
	GS	1.66	0.12	−12.49	2.12	0.9833	7.52
	CLTank	1.41	0.05	−24.73	2.00	0.9975	17.54
	MIII	1.57	0.08	−36.70	3.06	0.9931	23.38

**Table 6:** Vaping regimen influence on the efficiency in e-liquid vaporization (η) using the five tested atomizers calculated at each supplied power (P). A dash means that the MVE was out of the optimal heating regimen and is not considered.

	**Cub1**										
**P (W)**	**9**	**12**	**15**	**18**	**21**	**24**	**27**	**30**	**33**	**36**	**39**
η (low regimen)	10%	23%	30%	33%	35%	36%	39%	-	-	-	-
η (intense regimen)	-	23%	35%	45%	48%	54%	59%	59%	62%	64%	64%
	**Nauti**					**GSAir**					
**P (W)**	**6.1**	**8.9**	**12.3**	**16.2**	**20**	**8**	**12**	**16**	**20**	**24**	
η (low regimen)	17%	34%	43%	45%	46%	15%	36%	40%	42%	-	
η (intense regimen)	-	-	29%	53%	74%	-	23%	51%	55%	58%	
	**MIII**					**CLTank**					
**P (W)**	**30**	**35**	**40**	**45**	**50**	**15**	**25**	**35**	**45**	**50**	
η (low regimen)	15%	20%	22%	24%	25%	3%	13%	17%	19%	19%	
η (intense regimen)	18%	28%	35%	37%	44%	-	20%	38%	45%	47%	
